# Medical Extracts

**Published:** 1885-09

**Authors:** 


### Medical Extracts.

---

### Chorea: Treatment by Chloral.

Mons. A. Joffroy, of the Hopital des Enfants-Malades, strongly recommends the systematic administration of chloral in the treatment of chorea. He administers this drug in sufficient doses to induce sleep, which is only broken twice in the twenty-four hours for the administration of food. This treatment has been methodically continued during fifteen days, a month, six weeks, or two months, up to complete recovery. The method succeeds in ameliorating the symptoms and in procuring a prolonged sleep. It is of importance to regulate carefully the dose of the medicine. Above the age of ten years, he ordinarily gives four grammes of chloral in three doses after meals: a gramme in the morning, a gramme at noon, and two grammes at night. For infants, from six to eight years, the quantity should not exceed three grammes. In all cases it is necessary to graduate the doses in such a way that the artificial sleep be always surely obtained a quarter of an hour after the administration of the dose, at least after the strongest dose, which is given at night. This mode of treatment should be continued with regularity up to the time when the choreic jactitation has been completely suppressed, and when the cure is complete. The chloral is given to the children in combination with currant jelly, and is prepared as follows: A concentrated aqueous solution of pure chloral (4 grammes to 1 of water) is combined with currant jelly, the mixture containing 1 gramme of the drug in each dessert-spoonful. The little patients take this preparation very easily in finishing their meals. In the majority of cases this treatment is found to be efficacious and sufficient. —*Le Progrès Médical*, 13th June, 1885.

### Some Indications for Antipyrine.

Mr. G. Daremberg communicates to the Academy of Medicine a paper on "Some Indications for Antipyrine, especially in the Pyrexia of Tuberculosis."

He gives the following rule for its administration: To give the first dose of one gramme of antipyrine before the commencement of the fever—that is to say, before the thermometer reaches 37°6; then to take another gramme every time that the thermometer has risen more than three tenths in one hour. If, however, the thermometer has risen only two tenths in an hour, it is necessary to give a dose if the patient ought to take a meal. It is right to allow an interval of one hour between a dose of the medicine and a meal; otherwise the digestion is damaged.

The drug may be continued for a long time in doses of 4 to 6 grammes daily with no disadvantage, in doses of 1 gramme at such times as the thermometer indicates, and principally one hour before meals.—*Bulletin de l'Académie de Médicine*, No. 21, 1885.

### Thalline.

A derivative of quinoline, discovered by Dr. v. Jaksch, de Vienna, has a more agreeable action than kairine, and is more active than antipyrine. As a single dose, 4, 8, 12 grains were given. Thalline has the advantages over antipyrine of smaller dose, a less disagreeable taste and smell, and of rarely producing vomiting. Antipyrine has, on the contrary, the advantages over thalline of much longer duration of action, the absence of shivering, slowness of pulse in proportion to fall of temperature, and a more favourable action on the joints in acute articular rheumatism.—*Le Progrès Médicale*, May 16th, 1885.

M. Jaccoud gives his experience on the action of thalline, comprising a summary of the results of 43 administrations in 11 patients—24 administrations to cases of typhoid, 17 to cases of febrile tuberculosis, once to a case of pneumonia, and once to a case of erysipelas of face—and he comes to the following conclusion: Thalline is an antipyretic which surpasses all other antithermic agents. By the administration of 5 or 10 centigrammes every hour, one can maintain a permanent apyrexial condition in febrile cases. It is certain that in thalline we have the means of suppressing a febrile temperature for as long a time as we wish.—*Gazette des Hôpitaux*, June 25th, 1885.

### Contribution to the Pathology of Thomsen's Disease.

Professor Bernhardt, writing to the *Centralblatt für Nervenheilkunde* (No. 6, 1885), says: "The two following cases of Thomsen's disease are those of a youth 16½ years of age, and of his sister, a girl aged 18. Both patients say that the disease began when they were 4 or 5 years of age, and in the inferior extremities. In the case of the brother it has spread to other muscles, which have been attacked to the same extent as those of the legs, but in that of his sister the superior extremities have retained fairly normal power of movement. After a night's rest she does not feel stiff, and can leave her bed without any difficulty; but when she moves about much, she experiences the characteristic symptoms of the disease. If she gets up after sitting for some time, although she can rise from the chair quite well, as soon as she is on her feet she becomes stiff, and would fall, if touched ever so lightly; the first few steps she takes are stiff and awkward, after a few paces she walks better. By paying attention to her movements, and by doing her utmost to control the stiffness of the muscles by an effort of will, she succeeds in partially overcoming the difficulty of locomotion. All her other muscles are well developed.

"Her brother, a strongly-built young man, with athletic development of the muscles of arms and legs, exhibits the typical symptoms of the disease. The father and mother of these patients were blood relatives (cousins), also the grandparents. An aunt on the father's side was similarly affected. A sister of this aunt has married her cousin, and both the children of this marriage, a son and a daughter, have the disease. The son has married his cousin, and their child, a boy of 4 to 5 years of age, is showing the first symptoms of it."

### Clinical and Pathological Observations on Thomsen's Disease.

Professor Erb, of Heidelberg (*Neur. Centralblatt*, No. 13, 1885), says: "Clinical observation of the motor nervous systems and muscles of three brothers, aged about 14 or 15 years, who were affected with this disease, have shown that we have in reality to do with an abnormality in the form of the contraction of the muscles, and the striking indolence (prolonged latent period?) and the continuance of the contraction are characteristic. This 'myotonic contraction' can be overcome by *direct* action on the muscles (mechanical, faradic, or galvanic irritation); through the nervous system, on the other hand, only when a series of rapidly accumulating irritants, such as voluntary action, secondary faradic currents, or labile galvanic current, act on the nerves." The sum total of these changes in electric excitability, which in a characteristic manner differentiate them from the reaction of degeneration, of tetany, of muscular hypertrophy, &c., Erb

16

designates by the term "myotonic electric reaction." The examination of a piece of muscle excised from the biceps, showed considerable hypertrophy was present. The diameter of the muscular fibre varied from 24 to 180 $\mu$ (normal 25 to 65 $\mu$). More than 50 per cent. of the fibres were from 80 to 140 $\mu$ in breadth, whilst only 2 per cent. were under 40 $\mu$, and 8 per cent. were over 140 $\mu$. On measuring, for purposes of comparison, the biceps of a patient in good condition, who had died of an acute disease, the extreme limits of breadth were 12 and 80 $\mu$, 62 per cent. between 20 and 60 $\mu$, 7.5 per cent. under 20 $\mu$, and no fibres above 80 $\mu$. The fibres are of more rounded form, the angles being rounded off, and they are not pressed together in polygonal forms, as in the case of normal muscle. Erb also found that the nuclei of the sarcolemma were much increased in number, viz. 65, as compared with 18. The tranverse striae were very fine, often somewhat indistinct. At the end of his communication Erb expressly warns us against attaching too much value, at any rate prematurely, to the above abnormalities as supporting a theory of a myopathic origin of the disease.—*Deutsche Mediz.-Zeitung*, September 7th, 1885.

### Fatal Poisoning by Iodoform.

Dr. Schwarz relates a case in which Iodoform was used after a vaginal and perineal operation, in a woman who had previously shown signs of psychical disturbance, small strips of iodoformed gauze being placed in the vagina, and the powdered drug dusted on the wound, three or four grammes of the drug in all being used.

Sixty hours after the operation the woman refused food, drink, and medicine, because she thought that she would be poisoned; she then had an attack of acute mania, and hallucination followed this. Some hours after, all the iodoform still remaining on the wound was carefully removed, and she was kept under the influence of morphia and chloroform, with ice bladders to the head and neck. Her face was noted as being much flushed, the eyes prominent and glittering, the mouth and tongue dry and furred. On awaking from the narcosis the symptoms of mania returned, and she refused nourishment. The temperature rose, pulse strong, pupils contracted, thumbs strongly flexed on the palms, the neck stiff, and trismus ensued. On the 6th day after the operation the patient died. The post-mortem examination showed extensive disease of the left kidney, with calculi in the pelvis, and the left ureter was blocked. Right kidney fairly normal. Nothing peculiar about the wound. In the cerebrum, where the cortex and medulla are in contact, the former showed several reddish yellow spots, each of which was several millimetres in size.

Dr. Schwarz comes to the conclusion, from a study of the above case, that we ought to be very careful in using iodoform in old people, and especially where there is any predisposition to brain or kidney affection.

In connection with the case, the excretion of Iodine by the kidneys was noted by Dr. Harnack, and he found that on the first day after the toxic symptoms made their appearance, the urine removed by catheter contained 0·52 gramme of iodine per litre, of which amount 0·11 gramme was in the metallic form, the remainder in combination. The urine passed that day, after the iodoform had been removed from the wound, contained almost no iodine. The liver was quite free from iodine. The traces were found in the cerebrum, and the cerebellum contained one part of iodine in 5,000 parts of dried brain substance, which small amount, Dr. H. considers, may have a causal relationship to the psychical disturbances observed in iodoform poisoning.

In another case, which ended in recovery, Dr. H. found that the urine passed soon after the commencement of the nervous symptoms contained 0·23 gramme of iodine per litre, of which 0·175 gramme was in the form of the metal.—*Deutsche Mediz.-Zeitung*, August 13th, 1885.

### Lupus and Tuberculosis.

Dr. E. Schmiegelow, in discussing this subject, cites the following results of experiments by:

1. R. Koch, who found tubercle bacilli in lupous tissue, and produced generalized tuberculosis by inoculation of this tissue:

2. Pagentecher and Pfeiffer, who found the bacilli in the lupous nodules of conjunctivitis. Inoculation was performed into the anterior chamber of rabbits' eyes ; in five or six weeks typical tuberculosis of the iris and bacilli found in excised pieces of it:

3. Cornel and Leloir, who produced generalised tuberculosis by inoculating lupous tissue:

4. Dontrelepont, bacilli in connection with lupus, in 25 cases: as going far to settle the dispute as to the tubercular nature of lupus.

Schmiegelow considers that clinically nasal lupous polypi bear the same relation to lupous ulceration of the nose, as lupous granulations of the larynx and trachea bear to the tubercular ulcerations which are produced in these organs.

A practical point appears to be that the bacilli are few and far between in the lupous tissue, Koch having examined in one case 27, and in another 43 sections, before one was found.

Treatment of nasal lupus consisted of removal of the nodules by the sharp spoon, followed by galvano-cautery and solid nitrate of silver.—*Revue Mensuelle de laryngologie, &c.*, September, 1885.

### Injection of Japanese (India) Ink in the Preparation of Histological Specimens.

In anatomy and histology the injection of staining fluids into the blood and lymphatic vessels is indispensable for the investigation of their arrangement, distribution, and anastomoses. This useful process was originally practised by Berengarins Carpensis of Pavia and Bologna (1502-1527). He filled the vessels with a coloured or non-coloured fluid by blowing. After him the process was taken up by many others, such as R. Vieussens of Montpelliér (1641-1715), who used mercury; Joh. Swammerdan of Holland (1627-1680), who used melted wax, and Petrus Simon Rouhault, who used a kind of gelatine; but the object of their work was limited to anatomical purposes only. It was not until the present century that this art has been employed in histological work, for introductions of which we are indebted to Jos. Gerlach of Erlangen, who injected carmine into the vessels for microscopical investigation. From his time, the art has progressed materially. We have now many methods and many fluids for the same purpose; but I think the preparation of these fluids is rather complex, and some of them easily spoil by modifying the elements of the texture, or by infiltrating the tissues around the vessels. Some of them lose their colour by being kept any length of time. In 1879 I suggested that Japanese ink might be used for the same object, and I have had good results with it. I have made many beautiful preparations with it. The vessels of the brain, of the spinal cord, of the striated muscle, of the papillæ of the tongue, of the mucous membrane of the intestine, of the mucous membrane of the bladder, of the kidney, of the cavernous body of the penis, of the skin of the tip of the finger, of the palpebral conjunctiva, of the choroid of the iris. I have also tried it in the lymphatic vessels of the small intestine, of the retina, and of various other parts. It penetrates into the finest channel in every instance. In the small intestine it shows beautifully the lymphatic meshes of the submucous tissue, the lymphatic vessels of Lieberkühn's tube, the lymphatic cavity of the villi, and the lymphatic meshes of Peyer's patches. In the retina, it shows a network of black lines in the plane section, elliptical black spots in the transverse section, and slender spindle lines in the vertical section.

Ordinary Chinese or Japanese ink is sufficient. There is no danger of its spoiling in the warm season, or of its changing colour by being kept a long time. To prepare it, rub it slowly on the ink stone (*sudzuri ishi*) until it does not blot, or infiltrate into the surrounding surface when dropped upon Chinese paper. It may then be injected in the ordinary way with a syringe.—Dr. K. TAGUCHI (University of Tōkiō), in *Transactions of the Sei-i-Kwai*, Japan, May, 1885.

### Dr. Alexander on Excision of the Hip.

Dr. Alexander summarises his experience in the treatment of hip disease as follows:

1. That hip disease should in the earlier stages be treated by that absolute and perfect rest of the joint that we now appreciate so well and know so much better—thanks to Mr. H. O. Thomas—how to apply, without at the same time producing general debility of the system through the restraint and confinement necessary in former times to secure sufficient rest to the joint.

2. That this treatment, thoroughly and persistently carried out for a long period, will cure a very large percentage of cases of joint disease.

3. That unfortunately this treatment *cannot* or *is not* properly or persistently carried out amongst the poor, with whom a persistent attempt to carry it out after a certain stage of the disease has been reached only leads in many cases to a useless limb after many years, and probably in a majority to death, either during the process of cure or soon after, from the exhausting effects of the local disease, and not, as some erroneously think, from an inherited constitutional debility.

4. That many of these patients could be saved by excising the joint when a decided second stage of hip disease has been reached, and that excision is most safely and advantageously performed by severing the femur above the trochanters, clearing out the acetabulum, and maintaining the opposing bones so far apart that their surfaces can resume a healthy condition and the aperture between become filled up with fibrous tissue. By this means an excellent false joint is formed, or if the adhesions become too firm, a good stiff joint.

5. That the advent of the stage of the disease suitable for excision is indicated by the repeated formations of abscesses and sinuses round the joint. Then excision, as described, seems to me to offer better results, both locally and generally, than rest and waiting to see what will turn up.

6. That when the supra-trochanteric mode of excision cannot be performed with any chance of success, on account of the extensive nature of the hip disease, the alternative treatment comprises either continued expectancy or amputation, according to the age and condition of the patient.

7. That it is, however, a great mistake to imagine that all softened bone or infiltrated tissue should be cleared away by the operator. All he has got to do is to clear a space where the operations of Nature, in dealing with diseased or disabled tissues, can be carried out as easily and expeditiously as possible. The operator should remove all manifestly dead tissue ; but bone and tissue that the surgeon would be inclined to sacrifice as doubtful should be left alone, as these will, under the same skilful hands of Nature, be restored to health, or extruded harmlessly if only a passage for their extrusion be maintained.—*Liverpool Med.-Chirurgical Journal*, No. 9, July, 1885.

### What is the proper time to take Medicines?

The question whether a prescribed medicine should be taken before or after a meal is often put to the physician, and occasionally requires some special consideration. The medicines which act as local irritants, such as the salts of copper, iron, zinc, and arsenic, in large doses, are to be taken after a meal when the stomach is full, while small doses of medicines acting on the gastric terminations of the vagus ought to be taken before a meal. In some instances we have to consider chemical changes. Oxide and nitrate of silver, if intended to act locally on the gastric mucous membrane, must likewise be exhibited when the stomach is empty. It is not generally known, or at least observed, that iodine and its salts are to be administered on an empty stomach, as the presence of starch and acids, modifying or decomposing the preparations of iodine, would reduce or prevent their effect. The acids intended to affect the gastric juices are to be taken before a meal, in order to provoke an ample secretion of the gastric glands. If alkalies are to modify the gastric juices, they must be given during the meal; but if their absorption into the blood is desired, they ought to be ingested on an empty stomach, in order not to hinder the process of chymification by weakening the acids. Metallic salts, especially corrosive sublimate, likewise tannic acid, alcohol, and other drugs, modify or destroy the digestive power of pepsine, and are hence to be exhibited solely before meals. Small quantities of alcohol, as contained in the ordinary and medicinal wines, do not injuriously affect pepsine like the liquors rich in alcohol. Iron, phosphates, cod-liver oil, and similar medicines, may be taken during meal-time (*Bull. Gén. de Thérap.*).—*Therapeutic Gazette* (Detroit, U.S.A.), June 15, 1885.

### The Incompatibility of Chloral Hydrate with Potassium Bromide and Alcohol.

The decomposing of a prescription containing potassium bromide, chloral hydrate, tincture of opium and camphor, and syrup of ginger, led Professor F. M. Markoe (*Boston Medical and Surgical Journal*, July 23, 1885), to study the incompatibility of chloral. The various experiments which he made led him to the practical deduction that alcoholic preparations of all forms should not be prescribed with chloral hydrate, especially not with bromides of potassium and sodium, because if the solutions used are at all concentrated the chloral will separate as an alcoholate, float on the surface, and great risk will be incurred of giving a large overdose, the patient having received no caution as to shaking the contents of the bottle before taking the dose.—*Therapeutic Gazette*, August 15, 1885.

### The External Treatment of Night-sweats.

Nicolai (*Gazette Médicale de Paris*, June 6, 1885) has obtained very good results in the case of night-sweats of phthisical patients, and others, by the employment of 8 grammes of chloral dissolved in two tumblerfuls of a mixture of equal parts of brandy and water. Every evening before going to sleep the patients are washed over with a sponge soaked with this solution, and if that does not serve to control the sweating, the shirt in which the patient sleeps is soaked with the same solution and then dried. The effect of this treatment is claimed to be especially satisfactory in cases of children, not suffering from phthisis, in whom night-sweats are present. Sometimes four rubbings with this solution are sufficient to entirely arrest the night-sweats for several weeks. The tincture of belladonna is also highly recommended by Radakow for the suppression of the night-sweats of phthisis by external friction, with a mixture of 4 grammes of the tincture of belladonna with 30 grammes of water, the friction to be made about two hours before the ordinary onset of the sweating. The fluid is to be poured into the palm of the hand, and then rubbed over the entire body, with the exception of the head and the extremities, and the manipulation may be continued until the skin becomes quite moist. This treatment has been employed by Radakow in fifty cases, and he claims that it has not failed in a single instance, although sometimes localized sweatings appeared on the parts which had not been bathed with the tincture of belladonna.—*Therapeutic Gazette*, August 15, 1885.

### On the Influence of Agaricine on Perspiration.

Experiments instituted by Dr. Pibram, of Prague, with agaricine, on persons with an abundant or suppressed perspiration, extend our knowledge as to the action of this drug. His conclusions, as detailed in the *Zeitschrift für Therapie* of March 15, 1885, are as follows:

1. Agaricine is a scarcely ever failing remedy for the suppression of excessive perspiration, especially in phthisical patients.
2. In the physiological state agaricine sustains perspiration at a certain constant level.
3. In cases of suppression of copious perspiration by agaricine, the cutaneous and pulmonary discharges remain essentially unaltered.
4. The urinary organs discharging the surplus of liquid, and the diminished thirst decreasing ingestion of liquids, the hydrostatic equilibrium is thus re-established.
5. Moderate perspiration yields to a single $\frac{1}{6}$-grain dose, while profuse sweating requires repeated, equal, or increasing doses for its suppression.
6. The physiological action of the drug manifests itself five hours after its exhibition.
7. There are no undesirable after-effects attending its use.
8. Agaricine leads to improvement of the subjective state of phthisical patients by eliminating a constant strain of their flagging strength, without, of course, altering the pathological tissue-changes themselves.—*Therapeutic Gazette*, May 15, 1885.

### Neuropathic Sneezing.

Dr. Féré describes a case, which was under the care of Professor Charcot, of a girl 16 years of age, who had ancestors who had shown neuropathic tendencies, and who herself showed all the symptoms of hysteria. The sneezes followed each other very quickly, at the rate of 32 to 40 per minute, and in three weeks she sneezed 16,195 times. No nasal secretion at all was observed to succeed the attacks, and it was regarded as purely hysterical.—*Revue Mensuelle de Laryngologie, &c.*, August 1st, 1885.

